# Supplementary material for: FroM Superstring to Indexing: a space-efficient index for unconstrained k-mer sets using the Masked Burrows-Wheeler Transform (MBWT)
Source: Bioinform Adv. 2025 Nov 12;6(1):vbaf290. doi: 10.1093/bioadv/vbaf290 (PMC12800775; doi:10.1093/bioadv/vbaf290)
Supplement: vbaf290_Supplementary_Data [file vbaf290_supplementary_data.pdf]

Supplementary materials for FroM Superstring to Indexing: a space-efficient index for unconstrained k-mer sets using the Masked Burrows-Wheeler Transform (MBWT)

Ondej Sladký[1,2], Pavel Veselý[1], Karel Binda[3]

[1]Computer Science Institute, Charles University, Malostranské nám., 118 00 Praha, Czechia

[2]ETH Zurich, Rämistrasse, 8092 Zurich, Switzerland

[3]Inria, Irisa, Univ. Rennes, Campus de Beaulieu, 35042 Rennes, France

`ondra.sladky@gmail.com`, `vesely@iuuk.mff.cuni.cz`, `karel.brinda@inria.fr`

1 Experimental evaluation

All scripts, data tables, and additional information are available in the supplementary repository of our paper on `https://github.com/OndrejSladky/fmsi-supplement`. The experimental evaluation was done in the following five steps.

Step 1: Download of the source data. The *SARS-CoV-2* pangenome **sc2-pg** was downloaded from GISAID `https://gisaid.org/` (access upon registration, version 2024/05/20). The *E. coli* pangenome was obtained from the phylogenetically compressed 661k collection [2] as provided on `https://doi.org/10.5281/zenodo.4602622` [3]; all the genomes from the batches starting by '`escherichia_coli__`' were extracted and used subsequently. The dataset **ec-pg-all** represents the entire *E. coli* collection without quality filtering, whereas for **ec-pg-hq**, we retained only the high-quality genomes. The *S. pneumoniae* pangenome was downloaded from the RASE DB *S. pneumoniae* (`https://github.com/c2-d2/rase-db-spneumoniae-sparc/`). Metagenomic sample SRS063932 (Illumina raw reads) of human microbiome with accession SRX023459, denoted **mtg-ilm**, was downloaded from `https://www.hmpdacc.org/hmp/HMASM/`; we converted the fastq files into FASTA files by '`seqtk seq -A -C`'. Human RNA-seq Illumina raw reads with accession SRX348811, **rna-ilm**, and the human genome Illumina raw reads with accession SRX016231, **hg-ilm**, were downloaded using the prefetch tool from the SRA toolkit and then converted into the FASTA format by '`fastq-dump -split-3 -fasta`'. The Human genome assembly chm13v2.0, **hg-t2t**, was downloaded from `https://s3-us-west-2.amazonaws.com/human-pangenomics/T2T/CHM13/assemblies/analysis_set/chm13v2.0.fa.gz`. The MiniKraken datasets (4GiB and 8GiB) were downloaded from `https://ccb.jhu.edu/software/kraken/`

Step 2: k-mer set preparation. The input files for the pangenomes, mtg-ilm, and hg-ilm reads were converted to unitigs by GGCAT v1.1.0 [4] by '`ggcat build -k {kmer-size} -m 200 -j 5 -s {min-freq} -o {preprocessed} {input_FASTA}`'. We used $k = 128$ and {min-freq}=1 for pangenomes and $k = 32$ and {min-freq}=2 for **mtg-ilm** and **hg-ilm**. No preprocessing was done for the HG assembly chm13v2.0 (hg-t2t). For MiniKraken datasets (4GiB and 8GiB), we dumped the 31-mers using Jellyfish 1.1.12. The obtained files with the k-mer sets were deposited on `https://zenodo.org/records/14722244`.

Step 3: Representation tailoring. Before indexing with SSHash [7] and FMSI, we used GGCAT ([4]; v1.1.1) and KmerCamel ([9]; v2.1.1), respectively, for preprocessing before indexing. The following commands were used (with k matching the k passed to the programs for indexing).

- For SSHash, we computed eulertigs, which is the shortest SPSS representation: '`ggcat build -k {kmer-size} -eulertigs -min-multiplicity 1 -memory 200 -threads-count 8 -o {eulertigs} {preprocessed}`'.
- For FMSI with the best available MS, including the optimization to max-one mask: '`kmercamel compute -k {kmer-size} -M {ms-maxone} -o {ms-minone} {preprocessed}`'. Alternatively, MS for FMSI was computed from eulertigs: '`kmercamel compute -S -k {kmer-size} -M {ms-maxone} -o {ms-minone} {eulertigs}`'.

Step 4: Generating k-mer queries. To generate queries, we simulated Illumina-like reads by WgSim ([5]; v0.3.1-r13) with the following command:'`wgsim -1{query_length} -d0 -S42 -s31 -e0 -r{mut_rate} -R0 -N{num_seqs} {wgsim_input} {queries} /dev/null`', where '`query_length`' was equal to k for isolated queries and to 300 for streaming queries, '`mut_rate`' was 0 for positive (isolated and streaming) queries and 0.1 for negative queries, '`num_seqs`' was set so that there are 10^6 k-mer queries overall (up to 300 less for streaming queries), and '`wgsim_input`' was dataset and query-type dependent; namely, for generating negative isolated queries, we used chromosome 1A of *T. aestivum* assembly GCF_018294505.1, downloaded from NCBI. We generated positive isolated and streaming queries from the original dataset or a reference genome in case of pangenomes (details provided in the supplementary repository). For input to the version of SBWT which does not store reverse-complement k-mers, we additionally duplicated every line of all query files and computed the reverse complements of the duplicated lines using '`seqtk seq -r`'.

Step 5: Construction and querying of the indexes We describe the commands used for the construction of indexes and running the queries. Time and memory for index construction are summarized in Table **S1**, including MS/SPSS computation of Step 3. The measured performance of the indexes is provided in **Tab. 2** and **Figs. 2-4**. Further information and experimental results can be found at `https://github.com/OndrejSladky/fmsi-supplement`.

- **FMSI** (`https://github.com/OndrejSladky/fmsi/`, v0.4.0, commit '`04c4580`'). FMSI has two variants, depending on the desired query type:
  - **Variant for membership queries:** index construction by '`fmsi index -k {kmer-size} {ms-maxone}`' and queries using '`fmsi query -k {kmer-size} -O -q {queries} {ms-maxone}`', additionally with '`-S`' in case of streaming queries.
  - **Variant for dictionary queries:** index construction by '`fmsi index -k {kmer-size} {ms-maxone}`' and lookup queries using '`fmsi lookup -k {kmer-size} -q {queries} {ms-minone}`', additionally with '`-S`' in case of streaming queries.

- **SBWT** ([1]; `https://github.com/algbio/SBWT`; commit '`b795178`'). We compiled SBWT separately for $k \leq 32$ and for $k \in (32, 64]$. The specific parameters were tuned based on the results provided in [1]. As the time-efficient variant, we use the default plain-matrix variant, with adding all reverse complements to the index. As the space-efficient variant, we use the rrr-split variant without reverse complements and queried both k-mer and its reverse complement.
  - **Fast variant:** index construction using '`sbwt build -k {kmer-size} -m 120 -t 8 -add-reverse-complements \\`
    `-i {prefix} -o {index-path}`', additionally with '`-no-streaming-support`' for isolated queries, and queries executed by '`sbwt search -i {index-path} -q {queries} -o /dev/null`'.
  - **Space-efficient variant:** index construction using
    '`sbwt build -variant rrr-split -k {kmer-size} -m 120 -t 8 -i {prefix} -o {index-path}`', additionally with `-no-streaming-support` for isolated queries, and queries executed by '`sbwt search -i {index-path} -q {queries_with_RCs} -o /dev/null`', always querying a k-mer and its reverse complement (RC).

- **SSHash** [7, 8] (`https://github.com/jermp/sshash`, commit '`d90ad37`'). A dictionary based on minimal perfect hashing of k-mers. We compiled SSHash separately for $k \leq 31$ and for $k \in [32, 63]$. Indexes were computed using '`sshash build -k {kmer-size} -m {M} -s {seed}` ', where we set the minimizer length to '`M`' $= \min\{\lceil \log_4 \#\ k\text{-mers} \rceil + 1, k - 2\}$ and used a random seed. Whenever the index construction failed, SSHash was executed again with a different random seed.

  - **Single variant:** index construction using '`sshash build -i {prefix} -k {kmer-size} -m {M} -o {index-path} -s {seed}`', where `prefix` is the input file with an SPSS of the k-mer set, and queries using '`sshash query -i {index-path} -q {queries}`'

- **CBL** [6] (`https://github.com/imartayan/CBL`, commit '`328bcc6`'), a very recent method based on smallest cyclic rotations of k-mers. The index was computed on canonical k-mers, to handle reverse complements, that is, we ran '`cbl build -c`'. CBL was compiled for every value of k separately, namely, we used '`RUSTFLAGS="-C target-cpu=native" K={kmer-size} PREFIX_BITS={pref_bits} \\ cargo +nightly build --release --examples --target-dir target.k_{kmer-size}`'

  where the pref_bits were set to 28 for $k \geq 15$, to 24 for $k = 13$, and to 20 for $k = 11$. We then used the following commands:

  - **Single variant:** index construction done using '`cbl build -c -o {index-path} {prefix}`', where `prefix` is the preprocessed dataset, and queries using '`cbl query {index-path} {queries}`'

2 Supplementary tables

Dataset (Subsampl. rate)	Tool	MS/SPSS tool	SPSS [min (GB)]	MS [min (GB)]	Index constr. [min (GB)]	Total [min] (max. GB)
ec-pg-hq (10%)	CBL	–	–	–	1.2 (1)	1.2 (1)
	SBWT-small	–	–	–	38.9 (20.3)	38.9 (20.3)
	SBWT-fast	–	–	–	83.2 (38.5)	83.2 (38.5)
	SSHash	GGCAT	4.2 (37)	–	0.7 (2.3)	4.9 (37)
	FMSI-memb	KmerCamel	–	14.2 (7.6)	2.7 (8.1)	16.9 (8.1)
	FMSI-memb	GGCAT + KmerCamel	4.2 (37)	47 (10.1)	2.7 (8.1)	53.9 (37)
	FMSI-dict	KmerCamel	–	17.4 (7.6)	3.2 (8.1)	20.6 (8.1)
	FMSI-dict	GGCAT + KmerCamel	4.2 (37)	57.6 (5.5)	3 (8.1)	64.8 (37)
ec-pg-hq (100%)	CBL	–	–	–	17.8 (5.8)	17.8 (5.8)
	SBWT-small	–	–	–	9.7 (80.9)	9.7 (80.9)
	SBWT-fast	–	–	–	20.8 (120)	20.8 (120)
	SSHash	GGCAT	14.5 (16.6)	–	1.9 (3)	16.4 (16.6)
	FMSI-memb	KmerCamel	–	23 (11)	3.8 (11.4)	26.9 (11.4)
	FMSI-memb	GGCAT + KmerCamel	14.5 (16.6)	14.3 (11)	3.8 (11.4)	32.6 (16.6)
	FMSI-dict	KmerCamel	–	27.8 (9.9)	4.5 (11.4)	32.3 (11.4)
	FMSI-dict	GGCAT + KmerCamel	14.5 (16.6)	12.7 (1.2)	6.2 (11.4)	33.4 (16.6)
hg-t2t 100%)	CBL	–	–	–	38.4 (101.1)	38.4 (101.1)
	SBWT-small	–	–	–	21.5 (49.9)	21.5 (49.9)
	SBWT-fast	–	–	–	39.5 (93.1)	39.5 (93.1)
	SSHash	GGCAT	30.5 (21.6)	–	14.6 (10.9)	45.1 (21.6)
	FMSI-memb	KmerCamel	–	52.7 (39)	18.5 (44.2)	71.2 (44.2)
	FMSI-memb	GGCAT + KmerCamel	30.5 (21.6)	25.3 (39)	15.4 (44.2)	71.2 (44.2)
	FMSI-dict	KmerCamel	–	48.2 (36.9)	28.4 (44.2)	76.6 (44.2)
	FMSI-dict	GGCAT + KmerCamel	30.5 (21.6)	14.1 (1.7)	18.8 (44.2)	63.4 (44.2)
mtg-ilm (10%)	CBL	–	–	–	0.8 (0.8)	0.8 (0.8)
	SBWT-small	–	–	–	26.3 (13.6)	26.3 (13.6)
	SBWT-fast	–	–	–	52.7 (26)	52.7 (26)
	SSHash	GGCAT	2.8 (24)	–	0.5 (1.6)	3.3 (24)
	FMSI-memb	KmerCamel	–	10.4 (4.9)	1.8 (5.5)	12.1 (5.5)
	FMSI-memb	GGCAT + KmerCamel	2.8 (24)	28.6 (6.3)	1.8 (5.5)	33.1 (24)
	FMSI-dict	KmerCamel	–	11.1 (4.9)	2.1 (5.5)	13.2 (5.5)
	FMSI-dict	GGCAT + KmerCamel	2.8 (24)	35.3 (3.6)	2 (5.5)	40.1 (24)
mtg-ilm (100%)	CBL	–	–	–	3.2 (3.7)	3.2 (3.7)
	SBWT-small	–	–	–	6.6 (5.9)	6.6 (5.9)
	SBWT-fast	–	–	–	10.4 (11.7)	10.4 (11.7)
	SSHash	GGCAT	4.6 (7.3)	–	1.2 (2.2)	5.8 (7.3)
	FMSI-memb	KmerCamel	–	12.7 (5.8)	2.5 (7.6)	15.2 (7.6)
	FMSI-memb	GGCAT + KmerCamel	4.6 (7.3)	9.6 (5.8)	2.5 (7.6)	16.8 (7.6)
	FMSI-dict	KmerCamel	–	15 (5)	2.9 (7.6)	18 (7.6)
	FMSI-dict	GGCAT + KmerCamel	4.6 (7.3)	9.6 (0.7)	2.9 (7.6)	17.1 (7.6)

Table S1: Construction time and memory usage for superstrings and indexes. Construction CPU time (usr+sys, minutes) and memory usage (GB, in parentheses) for selected datasets with $k = 31$. SPSS are optimal simplitigs (eulertigs) computed by GGCAT [4]; MS is computed using the global greedy algorithm of KmerCamel [9], either from preprocessed input or from SPSS. GGCAT and SBWT were run on 8 threads; the other tools support only single-threaded construction.

References

[1] J. N. Alanko, S. J. Puglisi, and J. Vuohtoniemi. Small searchable κ-spectra via subset rank queries on the spectral Burrows-Wheeler transform. In *ACDA '23*. SIAM, 2023.

[2] G. A. Blackwell, M. Hunt, K. M. Malone, L. Lima, G. Horesh, B. T. F. Alako, N. R. Thomson, and Z. Iqbal. Exploring bacterial diversity via a curated and searchable snapshot of archived dna sequences. *PLOS Biology*, 19(11), 2021.

[3] K. Břinda, L. Lima, S. Pignotti, N. Quinones-Olvera, K. Salikhov, R. Chikhi, G. Kucherov, Z. Iqbal, and M. Baym. Efficient and robust search of microbial genomes via phylogenetic compression. *Nature Methods*, (4):692–697, 2025.

[4] A. Cracco and A. I. Tomescu. Extremely fast construction and querying of compacted and colored de Bruijn graphs with ggcat. *Genome Research*, 2023.

[5] H. Li. wgsim. `https://github.com/lh3/wgsim`, 2011.

[6] I. Martayan, B. Cazaux, A. Limasset, and C. Marchet. Conway–Bromage–Lyndon (CBL): an exact, dynamic representation of k-mer sets. *Bioinformatics*, 40:i48–i57, 2024.

[7] G. E. Pibiri. Sparse and skew hashing of K-mers. *Bioinformatics*, 38(Supplement_1), 2022.

[8] G. E. Pibiri. On weighted k-mer dictionaries. *Algorithms for Molecular Biology*, 18(1), 2023.

[9] O. Sladký, P. Veselý, and K. Břinda. Masked superstrings as a unified framework for textual k-mer set representations. *bioRxiv*, 2023.02.01.526717, 2023.
